# The association between headphones use during study and concentration among medical student at King Khalid University: A cross-sectional study

**DOI:** 10.1097/MD.0000000000041655

**Published:** 2025-02-21

**Authors:** Ayoub Ali Alshaikh, Ali Mohammed AlAmri, Meteb Ahmad Albraik, Khaled Abdulwahab N. Amer, Ali Abdullah A. Alqahtani, Rayan Mohammed S. Almugharrid, Abdulmohsin Mohammed S. Alzuhari, Omair Mohammed O. Alshahrani, Syed Esam Mahmood

**Affiliations:** a Department of Family & Community Medicine, College of Medicine, King Khalid University, Abha, Saudia Arabia; b Family Medicine and Diabetes Consultant, Joint Program of Family Medicine, Southern Region, Abha, Saudi Arabia, Ministry of Health, Abha, Saudi Arabia; c Abha Health Sector, General Directorate of Health Affairs, Aseer, KSA; d College of Medicine, King Khalid University, Abha, Saudi Arabia.

**Keywords:** academic performance, concentration, headphones, medical students, study habits

## Abstract

Headphones are commonly used by students to enhance concentration, particularly in high-pressure academic settings. However, the impact of headphone use on concentration and the potential health risks associated with prolonged use remain underexplored. This study examines the relationship between headphone use during study sessions and its effect on concentration among medical students. A cross-sectional survey was conducted among 359 medical students at King Khalid University, Abha, Saudi Arabia. The survey assessed demographic characteristics, headphone usage habits, perceived concentration levels, advantages and disadvantages of headphone use, and anxiety status. Among participants, 40% (n = 143) reported using headphones while studying. Headphone use was more prevalent among females (59.2%), sixth-year students (33.1%), and those from higher-income families (43.7%). Logistic regression analysis revealed significant associations between demographic factors and headphone use. Students using headphones for longer durations reported higher concentration levels and improved learning outcomes. Key advantages included blocking external distractions (53.5%), improving focus (47.9%), and enhancing study enjoyment (39.6%), while disadvantages included reduced situational awareness (52.8%). Higher volume levels correlated with increased concentration, with 50% of high-volume users reporting very high concentration. Additionally, family income level was significantly associated with concentration, with students from higher-income families exhibiting greater focus. Headphone use during study sessions is associated with increased concentration among medical students. Understanding usage patterns and their effects on academic performance is crucial for optimizing study environments and promoting effective learning strategies in medical education.

## 1. Introduction

Humans constantly interact with environmental stimuli through their sensory organs, significantly shaping their emotions, thoughts, and decision-making processes.^[1]^ Among these stimuli, sound plays a crucial role in influencing perception and emotional experiences in daily life. With the advancement of modern technology, devices such as mobile phones, computers, and music players have become significant sources of sound, delivered either through speakers or headphones. Headphones, in particular, provide a more focused and immersive auditory experience by minimizing external disturbances compared to speakers.^[2]^ The growing dependence on headphones has resulted in a significant rise in their usage. A study conducted in Saudi Arabia found that 80% of young individuals use headphones for music listening, with daily usage averaging between 0.5 to 2 hours.^[3]^ While headphones can enhance concentration and minimize distractions, it is important to note that prolonged exposure to high noise levels may result in sensorineural hearing loss, often referred to as noise-induced hearing loss.^[4]^

Beyond auditory effects, research has explored the cognitive and psychological impact of headphone use. Jurkovic et al showed that memory recall and retention increased while listening to headphones.^[5]^ Kulawiak study highlighted the academic advantages of noise-canceling headphones, demonstrating their potential to reduce distractions and enhance focus during class.^[6]^ Similarly, Townsend et al found that noise-canceling headphones could lower anxiety levels, thereby improving concentration during study sessions.^[7]^ The psychological stress induced by the coronavirus disease-19 pandemic, particularly among medical students, has further underscored the necessity of creating an optimal study environment to enhance focus and mental well-being.^[8–12]^

Additionally, Wang et al emphasized the importance of health education interventions to mitigate hearing health risks associated with headphone use.^[13]^ This raises concerns about balancing the benefits of headphones in improving concentration with the potential health risks associated with prolonged usage. The use of headphones can change an individual’s auditory environment and perception of the world by offering a different sound experience and influencing our capacity to engage with others.^[14]^ The Beck Anxiety Inventory is a well-established scale for assessing anxiety severity, and used to identify any significant correlations between how often and in what contexts individuals use headphones, whether for music, podcasts, or other audio content, and their reported anxiety levels.^[15]^ Utilizing noise reduction headphones and a personal electronic device during the cast removal process decreases patient anxiety.^[16]^

The findings from these studies could provide valuable insights into the dual role of headphone use as a potential coping mechanism for anxiety, as well as its possible contribution to feelings of isolation or heightened anxiety in specific situations. By analyzing the data gathered through the Beck Anxiety Inventory, researchers can gain a deeper understanding of the psychological effects of headphone use and its implications for mental health. This research is particularly pertinent in contemporary society, where headphone use is widespread, making it essential to comprehend its impact on emotional well-being.

Given the demanding nature of medical education, where students often face high cognitive loads and extended study hours, understanding the effects of headphone use on concentration is particularly relevant. This study aims to explore the relationship between headphone usage and concentration levels among medical students, assessing its advantages, potential drawbacks, and long-term implications. The study emphasizes the health risks linked to headphone use, aiming to inform educational institutions and policymakers about the need for safe listening practices. By increasing awareness, it advocates for the creation of guidelines and educational programs to safeguard students’ auditory health. Additionally, the study may stimulate further research into safer audio technologies that enhance learning while minimizing health risks. Ultimately, this knowledge seeks to foster academic environments that prioritize both student well-being and educational success.

## 2. Subject and methods

### 2.1. Study design

This study adopted a cross-sectional survey design to evaluate the association between headphone use during study sessions and concentration levels among medical students at King Khalid University, Abha, Saudi Arabia.

### 2.2. Study participants and sample size calculation

The study focuses on medical students, who face distinct academic pressures and psychological stressors that influence their study habits and coping mechanisms. It highlights that the rigorous demands of medical education often lead students to use techniques, such as wearing headphones, to enhance concentration in stressful situations. Although they recognize potential health risks, students may still engage in prolonged headphone use, which could have negative health consequences. The research aims to examine the patterns of headphone usage in this demanding academic context and evaluate its impact on both concentration and health. The survey targeted medical students enrolled in various academic years, typically ranging in age from 18 to 26 years. This range reflects the standard age distribution of medical students, encompassing individuals in the early stages of their medical education (preclinical years) as well as those in the later, more demanding clinical years.

Using G*power, the minimum required sample size was 321, based on the following assumptions, alpha error of 0.05, power of 0.95%, size effect of 0.1, and 50% of medical students had impaired concentration during using headphones. This study involved medical students from King Khalid University. Participants were recruited using convenience sampling, and students of all academic years were invited to participate.

### 2.3. Data collection

From June to August 2023, data was collected using 2 methods: an online questionnaire and in-person interviews. The questionnaire was given electronically to participants via email and social media platforms, with face-to-face interviews conducted for those who preferred this manner. This dual approach helped us gather a more diverse and representative sample of medical students, enhancing the generalizability of our findings. To mitigate bias, we ensured that the same questionnaire was used in both formats, maintaining consistency in the data collection process.

The questionnaire was self-designed to address the specific objectives of our study, focusing on the relationship between headphone use and concentration among medical students. To ensure validity, the questionnaire underwent a thorough review process, including feedback from subject matter experts in medical education and survey design. Additionally, a pilot study was conducted to assess the feasibility, clarity, and reliability of the questionnaire items. Based on the feedback received during the pilot study, necessary refinements were made to enhance the quality and relevance of the questionnaire.

The questionnaire items address a variety of demographic and socioeconomic parameters relevant to understanding headphone use during study sessions and concentration levels among medical students. The first component of the questionnaire collected the following sociodemographic information: age, gender, current year of study, family size, and income status. Finally, this section evaluated the availability of a dedicated study place.

The second portion carefully evaluated numerous elements of headphone use during study sessions. First, participants were asked about their frequency and duration of headphone use. Following that, participants were asked about their listening choices, which included music, podcasts, audiobooks, and white noise or natural noises. The questionnaire also assessed participants’ perceived concentration levels when studying, both with and without headphones. Concentration levels were assessed using self-reported measures embedded within the questionnaire. While a formal scale for concentration was not employed, the structured questions provided a reliable subjective assessment of concentration changes associated with headphone use. Participants were also asked to reflect on any variations they noticed in their learning outcomes before and after using headphones, as well as their academic success as determined by GPA. Advantages and disadvantages associated with headphone use were also explored, along with participants’ volume preferences and feelings about the impact of headphone usage on their academic performance. Furthermore, participants were queried about their practices regarding breaks or restricting the duration of headphone use, as well as their favorite study environments.

The third section assessed the anxiety status by Hamilton Anxiety Rating Scale.^[17]^ The 14-item scale assesses both somatic anxiety (physical problems associated with anxiety) and psychic anxiety (mental agitation and psychological distress). Each item on the scale is defined by a set of symptoms. A total score range of 0 to 56 is assigned to each item, with a range of 0 (not present) to 4 (severe). A score of <17 denotes light severity, 18 to 24 mild to moderate severity, and 25 to 30 moderate to severe. Before data collection, a pilot study was undertaken to assess the survey’s feasibility and accessibility. Each participant in the pilot study was asked to invite a minimum of 4 other individuals to participate in the main survey, thereby expanding the study sample and ensuring sufficient representation. The goal was to evaluate the time taken to complete the questionnaire and the clarity of the questions. This technique allowed researchers to calculate the average time necessary to complete the survey and the response rate. By determining the average completion time, we ensured the questionnaire was concise, clear, and accessible to all participants, regardless of the mode of administration.

### 2.4. Data analysis

Descriptive statistics were utilized to summarize participants’ demographic characteristics and headphone usage behaviors. Inferential statistics, such as chi-square testing and regression analysis, were used to investigate the relationship between headphone use during study sessions and concentration levels.

### 2.5. Ethical considerations

Before data collection began, the Institutional Review Board of King Khalid University provided ethical approval (ECM#2023-2801). All participants provided informed consent before being included in the study, and responses were kept anonymous throughout the research procedure.

## 3. Results

A total of 359 medical students participated in our study. The mean age of study participants was 19 years old. Approximately 66.4% of the participants were females, 29.1% in their sixth degree, and 42.6% thief family income status was more than 20,000 SAR. While 58.5% had 6 to 8 family members, 72.3% had a room for study.

Of the surveyed participants, 143 (40%) used headphones during study sessions. The participants who used headphones were more likely to be females (59.2%), in their sixth degree (33.1%), and with more than 20,000 SAR family income (43.7%). Also, they were more likely to have 6 to 8 family members (23.2%) and a room for study (66.2%).

**Figure 1.**
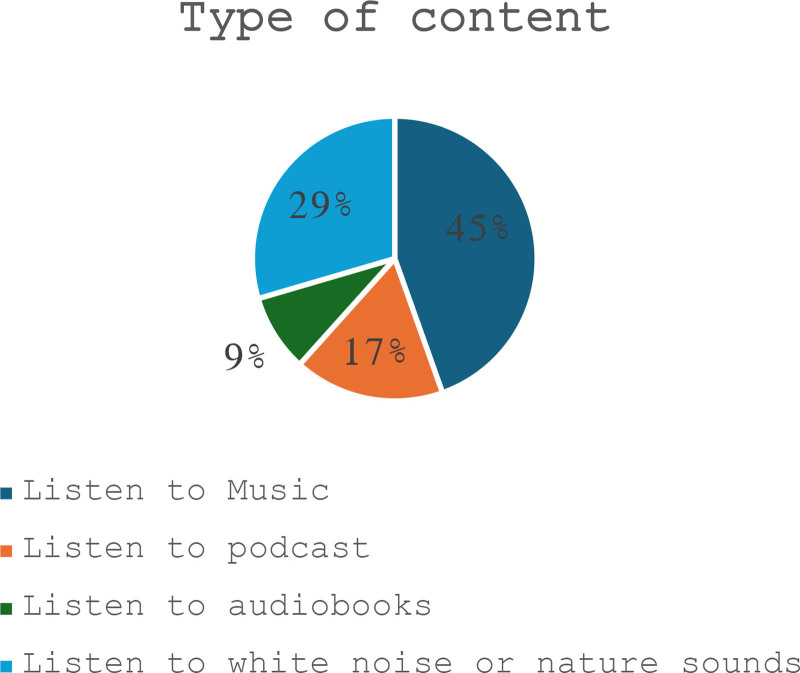
Type of content the student listens to while studying.

Comparing to those who did not use headphones, gender and having a room for study significantly affect the prevalence of using headphones (*P*-value = .019, *P*-value = .037) respectively (Table 1). A 45% of the participants listened to music (Fig. 1).

**Table 1:** General characteristics of headphone users and control group.

Characteristics		Using headphones		*P*-value
	Total	No	Yes	
N	359	215 (60%)	143 (40%)	
Age (year), mean (SD)	19 (8.4)	19 (8.2)	19.6 (8.6)	.49
Gender				**.019**
Male	120 (33.6%)	62 (28.8%)	58 (40.8%)	
Female	237 (66.4%)	153 (71.2%)	84 (59.2%)	
Current year of study				.12
First year	7 (2.0%)	4 (1.9%)	3 (2.1%)	
Second year	23 (6.4%)	11 (5.1%)	12 (8.5%)	
Third year	83 (23.2%)	55 (25.6%)	28 (19.7%)	
Fourth year	56 (15.7%)	34 (15.8%)	22 (15.5%)	
Fifth year	56 (15.7%)	41 (19.1%)	15 (10.6%)	
Sixth year	104 (29.1%)	57 (26.5%)	47 (33.1%)	
Intern	28 (7.8%)	13 (6.0%)	15 (10.6%)	
Family members				.78
<6 members	69 (19.3%)	44 (20.5%)	25 (17.6%)	
6–8 members	209 (58.5%)	125 (58.1%)	84 (59.2%)	
More than 8 members	79 (22.1%)	46 (21.4%)	33 (23.2%)	
Income status				.81
<5000 SAR	17 (4.8%)	12 (5.6%)	5 (3.5%)	
5001–10,000 SAR	58 (16.2%)	36 (16.7%)	22 (15.5%)	
10,001–20,000 SAR	129 (36.1%)	76 (35.3%)	53 (37.3%)	
>20,000 SAR	153 (42.9%)	91 (42.3%)	62 (43.7%)	
Room for study				**.037**
No	99 (27.7%)	51 (23.7%)	48 (33.8%)	
Yes	258 (72.3%)	164 (76.3%)	94 (66.2%)	

*P*-values <.5 are significant.

Table 2 shows level of concentration during studying among participants who used headphones. Level of concentration was low within 12% of the participants, 35% with moderate concentration, 44% with high concentration, and 8.3% with very high concentration.

**Table 2:** Concentration level among participants who used headphone.

Factor	Total	Level of concentration				
		Low	Moderate	High	Very high	p-value
N	143	18 (12%)	50 (35%)	63 (44%)	12 (8.3%)	
Frequency of using headphones while studying						**<.001**
Rarely or never	14 (9.8%)	7 (41%)	3 (6%)	3 (5%)	0 (0%)	
Occasionally (1–2 times a week)	41 (28.7%)	8 (47%)	18 (36%)	13 (36%)	2 (17%)	
Regularly (3–4 times a week)	36 (25.2%)	1 (6%)	16 (%)	16 (25%)	3 (25%)	
Always	52 (36.4%)	1 (6%)	31 (49%)	31 (49%)	7 (58%)	
how long do you use headphones during a study session?						**.003**
<15 minutes	4 (2.8%)	2 (12%)	2 (4%)	0 (0%)	0 (0%)	
15–30 minutes	10 (7.0%)	5 (29%)	3 (6%)	2 (3%)	0 (0%)	
30 minutes–1 hour	24 (16.8%)	4 (24%)	11 (22%)	8 (13%)	1 (8%)	
1–2 hours	37 (25.9%)	4 (24%)	13 (26%)	17 (27%)	2 (17%)	
>2 hours	68 (47.6%)	2 (12%)	21 (42%)	36 (57%)	9 (75%)	
Listen to Music	86 (59.7%)	10 (63%)	31 (62%)	39 (62%)	5 (42%)	.63
Listen to podcast	33 (22.9%)	4 (25%)	17 (34%)	9 (14%)	3 (25%)	.17
Listen to audiobooks	17 (11.8%)	1 (6%)	6 (12%)	6 (10%)	4 (33%)	.19
Listen to white noise or nature sounds	57 (39.6%)	5 (31%)	18 (36%)	31 (49%)	3 (25%)	.30
Difference in your learning outcomes when studying with headphones compared to w						**.009**
No	59 (41.3%)	9 (53%)	29 (58%)	19 (30%)	2 (17%)	
Yes	84 (58.7%)	8 (47%)	21 (42%)	44 (70%)	10 (83%)	
Advantages of using headphones						
Improve focusing	69 (47.9%)	1 (6%)	13 (26%)	44 (70%)	10 (83%)	**<.001**
Blocking external distraction	77 (53.5%)	9 (56%)	31 (62%)	33 (52%)	4 (33%)	.33
Enhanced enjoyment of studying	57 (39.6%)	5 (31%)	22 (44%)	27 (43%)	3 (25%)	.58
Disadvantages of using headphones						
Difficulty focusing	31 (21.5%)	11 (69%)	17 (34%)	3 (5%)	0 (0%)	**<.001**
Distraction	56 (38.9%)	11 (69%)	29 (58%)	14 (22%)	2 (17%)	**<.001**
Reduce awareness	76 (52.8%)	3 (19%)	24 (48%)	41 (65%)	7 (58%)	**.013**
Volume level						**<.001**
Low (barely audible)	17 (11.9%)	2 (12%)	7 (14%)	7 (11%)	0 (0%)	
Moderate (comfortable listening level)	76 (53.1%)	9 (53%)	30 (60%)	35 (56%)	2 (17%)	
High (loud enough to drown out background noise)	43 (30.1%)	5 (29%)	13 (26%)	19 (30%)	6 (50%)	
Very high (potentially damaging to hearing)	7 (4.9%)	1 (6%)	0 (0%)	2 (3%)	4 (33%)	
Feel about the impact of using headphones during studying on your academic perform						**<.001**
Negative	4 (2.8%)	2 (12%)	2 (4%)	0 (0%)	0 (0%)	
Neutral	57 (39.9%)	9 (53%)	33 (66%)	13 (21%)	2 (17%)	
Positive	59 (41.3%)	5 (29%)	14 (28%)	36 (57%)	3 (25%)	
Very positive	23 (16.1%)	1 (6%)	1 (2%)	14 (22%)	7 (58%)	
Take breaks or limit the duration of headphone use						.15
No	43 (30.1%)	6 (35%)	16 (32%)	14 (22%)	6 (50%)	
Yes	100 (69.9%)	11 (65%)	34 (68%)	49 (78%)	6 (50%)	
Where do you find it most conducive to study?						.16
Cafe or coffee shop	27 (18.9%)	1 (6%)	7 (14%)	13 (21%)	5 (42%)	
Home	90 (62.9%)	13 (76%)	33 (66%)	37 (59%)	7 (58%)	
Library	18 (12.6%)	3 (18%)	8 (16%)	7 (11%)	0 (0%)	
Outdoors	8 (5.6%)	0 (0%)	2 (4%)	6 (10%)	0 (0%)	
How would you describe your typical study environment?						.29
Quiet and free from distractions	84 (58.7%)	10 (59%)	34 (68%)	34 (54%)	5 (42%)	
Moderately quiet with occasional distractions	53 (37.1%)	6 (35%)	16 (32%)	26 (41%)	5 (42%)	
Noisy with frequent distractions	6 (4.2%)	1 (6%)	0 (0%)	3 (5%)	2 (17%)	

*P*-values <.5 are significant.

Participants who rarely headphones while studying were more likely to have low concentration (41%). While participants who always used headphones while studying were more likely to have very high concentration (58%). Participants who used headphones for more than 2 hours were more likely to have high concentration compared to those who used it for 15 to 30 minutes (57% vs 13%, *P*-value = .003). Participants who reported difference in learning outcomes when studying with headphones were more likely to have very high concentration comparing to those who reported no difference in studying outcomes (83% vs 17%, *P*-value = .009).

Blocking external distraction was the most reported advantage of using headphones among our participants (53.5%). This followed by improve focusing (47.9%) and enhanced enjoyment of studying (39.6%). In contrast, reduce awareness was the most reported disadvantage of using headphones (52.8%).

Volume level significantly affected the level of concentration. Participants who used high volume (loud enough to drown out background noise) more likely to have very high concentration (50%, *P*-value < .001). However, only 4.9% of our participants used very high (potentially damaging to hearing) volume. Also, participants who reported very positive impact of using headphones during studying were more likely to have very high concentration level compared to whose reported neutral impact (58% vs 17%, *P*-value < .001).

**Figure 2.**
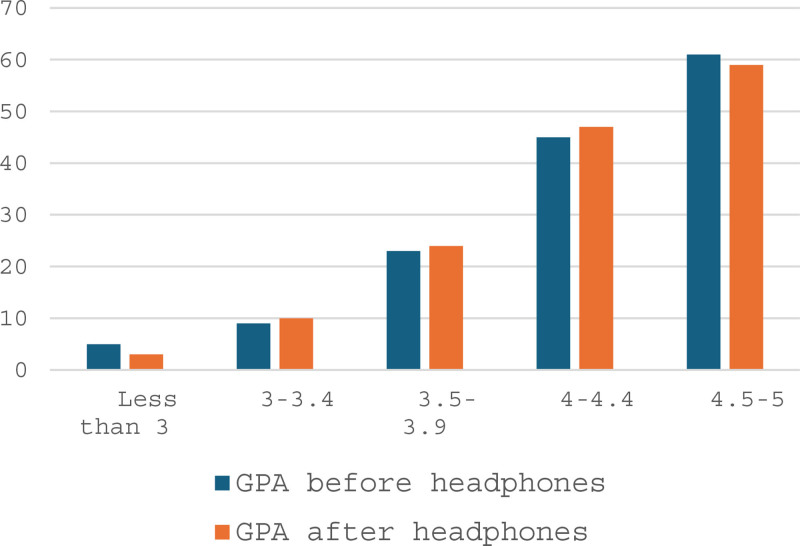
GPA before and after using headphones during studying.

Table 3 shows anxiety level among participants who used headphones. 65% of participants had mild level of anxiety, 22% had moderate to severe level, and 13% had mild to moderate severity. Participants with moderate to severe anxiety always used headphones during studying (37%), used it for more than 2 hours (30%). However, they reported moderate-high concentration with using headphones during studying (37%). Also, 60% of them reported difference in learning outcomes when studying with headphones. Using headphones while studying seems to help students with lower GPAs improve their performance (Fig. 2).

**Table 3:** Anxiety among participants who used headphone.

Factor	Total	Anxiety states			*P*-value
		Mild severity	Mild to moderate severity	Moderate to severe	
N	143	93 (65%)	19 (13%)	32 (22%)	
Frequency of using headphones while studying					.61
Rarely or never	14 (9.8%)	8 (9%)	2 (11%)	3 (10%)	
Occasionally (1–2 times a week)	41 (28.7%)	30 (32%)	3 (16%)	8 (27%)	
Regularly (3–4 times a week)	36 (25.2%)	20 (22%)	8 (42%)	8 (27%)	
Always	52 (36.4%)	35 (38%)	6 (32%)	11 (37%)	
How long do you use headphones during a study session?					.59
<15 minutes	4 (2.8%)	2 (2%)	0 (0%)	1 (3%)	
15–30 minutes	10 (7.0%)	6 (6%)	1 (5%)	3 (10%)	
30 minutes–1 hour	24 (16.8%)	14 (15%)	3 (16%)	7 (23%)	
1–2 hours	37 (25.9%)	24 (26%)	3 (16%)	10 (33%)	
>2 hours	68 (47.6%)	47 (51%)	12 (63%)	9 (30%)	
Listen to music	86 (59.7%)	53 (57%)	12 (63%)	21 (66%)	.66
Listen to podcast	33 (22.9%)	18 (19%)	5 (26%)	10 (31%)	.36
Listen to audiobooks	17 (11.8%)	13 (14%)	1 (5%)	3 (9%)	.50
Listen to white noise or nature sounds	57 (39.6%)	37 (40%)	6 (32%)	14 (44%)	.69
Very high	10 (7.0%)	8 (9%)	0 (0%)	2 (7%)	
Level of concentration during studying with headphones					.84
Very low	1 (0.7%)	1 (1%)	0 (0%)	0 (0%)	
Low	17 (11.9%)	8 (9%)	2 (11%)	6 (20%)	
Moderate	50 (35.0%)	32 (34%)	7 (37%)	11 (37%)	
High	63 (44.1%)	43 (46%)	9 (47%)	11 (37%)	
Very high	12 (8.4%)	9 (10%)	1 (5%)	2 (7%)	
Difference in your learning outcomes when studying with headphones compared to w					.82
No	59 (41.3%)	37 (40%)	9 (47%)	12 (40%)	
Yes	84 (58.7%)	56 (60%)	10 (53%)	18 (60%)	
Advantages of using headphones					
Improve focusing	69 (47.9%)	44 (47%)	12 (63%)	13 (41%)	.29
Blocking external distraction	77 (53.5%)	53 (57%)	10 (53%)	14 (44%)	.43
Enhanced enjoyment of studying	57 (39.6%)	38 (41%)	8 (42%)	11 (34%)	.79
Disadvantages of using headphones					
Difficulty focusing	31 (21.5%)	16 (17%)	5 (26%)	10 (31%)	.21
Distraction	56 (38.9%)	29 (31%)	8 (42%)	19 (59%)	**.018**
Reduce awareness	76 (52.8%)	52 (56%)	12 (63%)	12 (38%)	.12
Volume level					.99
Low (barely audible)	17 (11.9%)	11 (12%)	2 (11%)	3 (10%)	
Moderate (comfortable listening level)	76 (53.1%)	51 (55%)	9 (47%)	16 (53%)	
High (loud enough to drown out background noise)	43 (30.1%)	26 (28%)	7 (37%)	10 (33%)	
Very high (potentially damaging to hearing)	7 (4.9%)	5 (5%)	1 (5%)	1 (3%)	
Feel about the impact of using headphones during studying on your academic perform					.44
Negative	4 (2.8%)	1 (1%)	1 (5%)	2 (7%)	
Neutral	57 (39.9%)	37 (40%)	6 (32%)	14 (47%)	
Positive	59 (41.3%)	38 (41%)	9 (47%)	12 (40%)	
Very positive	23 (16.1%)	17 (18%)	3 (16%)	2 (7%)	
Take breaks or limit the duration of headphone use					.99
No	43 (30.1%)	28 (30%)	6 (32%)	9 (30%)	
Yes	100 (69.9%)	65 (70%)	13 (68%)	21 (70%)	
Where do you find it most conducive to study?					.48
Cafe or coffee shop	27 (18.9%)	21 (23%)	1 (5%)	5 (17%)	
Home	90 (62.9%)	55 (59%)	16 (84%)	19 (63%)	
Library	18 (12.6%)	11 (12%)	2 (11%)	4 (13%)	
Outdoors	8 (5.6%)	6 (6%)	0 (0%)	2 (7%)	
How would you describe your typical study environment?					.70
Quiet and free from distractions	84 (58.7%)	52 (56%)	10 (53%)	21 (70%)	
Moderately quiet with occasional distractions	53 (37.1%)	37 (40%)	8 (42%)	8 (27%)	
Noisy with frequent distractions	6 (4.2%)	4 (4%)	1 (5%)	1 (3%)	

Participants with moderate to severe anxiety reported neutral impact of using headphones on their academic performance (47%). Also, they prefer quiet and free from distractions environment for study.

In Table 4 logistic regression measured the associations between different risk factors and level of concentration with using headphones during studying. Among our participants, there was a significant association between concentration and family income level. Students with 5001 to 10,000 SAR family income had 15 times higher chance of having moderate to high concentration compared to those who had less family income 5000 SAR or less [OR: 15.75, 95% CI (1.57–157.6), *P*-value = .019].

**Table 4:** Association between level of concentration during headphones usage and different predictors.

Predictors		Concentration (low vs moderate-high)		
		OR	95% CI	*P*-value
Age		1.013	0.958–1.071	.646
Gender	Male	1		
	Female	0.701	0.247–1.99	.506
Current year of study	First year	1		
	Second year	5.5	0.234–128.968	.290
	Third year	3.125	0.226–43.021	.394
	Fourth year	3.166	0.215–46.726	.401
	Fifth year	1.375	0.096–19.643	.814
	sixth year	4.20	0.320–55.058	.274
	Intern	-		
Family members	<6	1		
	6–8	0.917	0.234–3.583	.901
	>8	0.989	0.2–4.878	.989
Income	5000 or less	1		
	5001–10,000	15.75	1.574–157.602	**.019**
	10,001–20,000	18.375	2.344–144.043	**.006**
	>20,000	8.833	1.290–60.473	**.026**
Room for study	No	1		
	Yes	1.304	0.471–3.610	.51

*P*-values <.5 are significant.

Also, students with –20,000 SAR family income had 18 times higher chance of having moderate to high concentration [OR: 18.37, 95% CI (2.34–144.04), *P*-value = .006] and those with income more than 20,000 SAR had 8 times higher chance of having moderate to high concentration compared to those who had less family income 5000 SAR or less [OR: 8.83, 95% CI (1.29–60.47), *P*-value = .026].

## 4. Discussion

The findings of this study revealed that 40% of medical students use headphones during study sessions, with females, sixth-year students, and those from higher-income families being more likely to adopt this practice. Participants reported that headphones enhanced concentration and improved learning outcomes when used for longer durations, primarily by blocking external distractions. However, a notable disadvantage was reduced environmental awareness, and higher volume levels were linked to greater concentration. Family income was also found to influence perceived concentration, with students from higher-income families reporting moderate to high concentration levels.

These findings align with existing literature, such as a study reporting a similar headphone usage rate of 37.3% among medical students or both talking and listening songs.^[18]^ However, other studies, such as that by Mohammad Poorasl et al, documented a significantly higher usage rate of 86.4% for listening to music, lectures, learning, and game playing,^[19]^ highlighting potential regional or demographic differences. Additionally, regional, cultural, and technological factors could account for variations in headphone usage rates. For example, access to high-quality headphones or noise-canceling devices may vary by location, as well as differences in academic environments or study habits. Despite their benefits, headphones pose risks, particularly related to hearing health. For example, Asghar et al reported that one-third of medical students using electroacoustic devices exhibited sensorineural hearing loss, with insert earbuds being the most commonly used devices.^[20]^ These findings emphasize the need for educational initiatives to promote safe listening habits, such as moderating volume levels, taking regular breaks, and undergoing periodic hearing assessments to mitigate potential adverse effects.

While this study found a positive association between headphone use and concentration, the evidence supporting this link remains inconclusive. A systematic review of 13 studies on noise-canceling headphones indicated limited and inconsistent findings regarding their effectiveness in improving academic performance.^[6]^ The lack of robust evidence, small sample sizes, and absence of replication studies suggest that more comprehensive research is required to establish clear guidelines on the role of headphones in enhancing focus and academic success.

Interestingly, this study did not find a significant association between headphone use and anxiety levels. This contrasts with findings from research using Korea National Health and Nutrition Examination Survey data, which reported that earphone users were more likely to experience tinnitus and anxiety symptoms compared to non-users.^[21]^ Such discrepancies could stem from differences in study populations, cultural contexts, or methodologies. These variations underscore the importance of exploring the multifaceted relationship between headphone use and psychological well-being through further research.

Overall, the study highlights the dual role of headphones as tools to enhance concentration and mitigate distractions, while also posing potential health risks if not used responsibly. Headphones can enhance concentration by blocking distractions and improving the learning experience, particularly for students with specific needs. However, they also pose significant risks to hearing health, primarily due to high volume levels, prolonged use, and the type of headphones used. To strike a balance, it is essential to promote safe listening practices, such as keeping the volume below 60% and taking regular breaks.^[22]^ By raising awareness about these risks and advocating for moderation, we can help students enjoy the advantages of headphones while safeguarding their auditory health. Promoting awareness of safe headphone practices and conducting large-scale studies to better understand their long-term impacts is crucial for optimizing their use in academic settings. These efforts would ensure that students can maximize the benefits of headphones without compromising their hearing health or psychological well-being.

The outcomes of the study highlight the complex relationship between headphone use, concentration, and health risks. While the health risks of headphone use, such as hearing loss and tinnitus, are well-documented, the concentration-enhancing effects are still debated in the literature. Our findings suggest that headphone use may improve focus by blocking external distractions, but this is not conclusive evidence of causality. Our study highlights a potential association rather than establishing a definitive causal relationship.

### 4.1. Strengths and limitations

This study utilized a mixed mode of data collection, incorporating online questionnaires and face-to-face interviews, to explore headphone usage and its impact on concentration among medical students. With a sample size of 359 participants, the study offers valuable insights into factors influencing headphone use and concentration. However, it is limited by self-reporting bias, its cross-sectional design, and restricted generalizability. Additionally, confounding variables like sleep quality and external stressors were not accounted for, warranting further exploration and careful interpretation of the findings. Future studies could benefit from larger sample sizes to enhance precision further and explore the nuanced relationships between headphone use and concentration. This will help in refining the effect size and solidifying the generalizability of findings.

### 4.2. Implications of the study

The findings have significant implications for medical education and student well-being. They highlight the widespread use of headphones among medical students, underscoring the need for personalized interventions to promote responsible use and mitigate potential risks. Educational institutions can integrate safe listening practices into their curricula, enabling students to make informed decisions about headphone use.

Additionally, educators might explore innovative teaching strategies that leverage headphone technology to enhance focus and reduce distractions. Students can apply the insights to refine their study habits and improve learning outcomes. Raising awareness of hearing health issues and promoting responsible headphone use are critical steps. By prioritizing student well-being and providing resources for auditory health, institutions can create a supportive learning environment.

## 5. Conclusions

This study provides valuable insights into the relationship between headphone use during study sessions and concentration levels among medical students. Headphone use was found to be influenced by demographic factors such as gender, academic year, and household income. It was associated with improved focus, particularly for students who used headphones for extended periods and observed positive changes in learning outcomes. While headphones helped minimize external distractions, they also posed challenges like reduced situational awareness, underscoring the need for balanced, and mindful usage. Furthermore, the association between higher volume levels and increased focus highlights the importance of practicing safe listening techniques. Headphones can significantly enhance concentration by minimizing external distractions and fostering a personalized auditory environment. This is particularly beneficial in noisy or shared study settings, such as libraries or dormitories, where ambient noise can hinder focus. The use of noise-canceling headphones has been suggested in previous studies to help maintain concentration by reducing auditory distractions, although further research is necessary to fully validate these findings. In conclusion, while headphones can enhance focus and improve mood for students during study sessions by mitigating external distractions such as background noise, they also present certain drawbacks. These include potential discomfort, diminished awareness of one’s surroundings, and possible health risks. Therefore, it is essential for students to weigh the benefits against the disadvantages when deciding to use headphones as a study aid.

The study’s findings call for several recommendations. Educational institutions should focus on raising awareness about both the benefits and risks of headphone use, encouraging safe listening habits and effective study practices. Academic support programs can help students optimize their learning environments and incorporate these strategies into daily routines. Future research should investigate the long-term effects of headphone use on academic performance and hearing health. Health education campaigns are essential to emphasize hearing health risks and promote cautious headphone use. Finally, establishing policies for headphone use in educational settings can foster a positive learning environment while supporting student well-being.

## Author contributions

**Conceptualization:** Ayoub Ali Alshaikh.

**Investigation:** Ayoub Ali Alshaikh, Ali Mohammed AlAmri, Meteb Ahmad Albraik, Khaled Abdulwahab N. Amer, Ali Abdullah A. Alqahtani, Rayan Mohammed S. Almugharrid, Abdulmohsin Mohammed S. Alzuhari, Omair Mohammed O. Alshahrani.

**Supervision:** Ayoub Ali Alshaikh.

**Writing – original draft:** Ayoub Ali Alshaikh.

**Writing – review & editing:** Syed Esam Mahmood.
